# TNFα-induced Up-regulation of *Ascl2* Affects the Differentiation and Proliferation of Neural Stem Cells

**DOI:** 10.14336/AD.2018.1028

**Published:** 2019-12-01

**Authors:** Zhongfeng Liu, Xuan Wang, Kewen Jiang, Xunming Ji, Y. Alex Zhang, Zhiguo Chen

**Affiliations:** ^1^Cell Therapy Center, Beijing Institute of Geriatrics, Xuanwu Hospital, Capital Medical University, and Key Laboratory of Neurodegeneration, Ministry of Education, Beijing, China; ^2^Center of Neural Injury and Repair, Beijing Institute for Brain Disorders, Beijing, China; ^3^Center of Parkinson's Disease, Beijing Institute for Brain Disorders, Beijing, China; ^4^Department of Neurology, the Children’s Hospital School of Medicine, Zhejiang University, Hangzhou, China; ^5^Department of Neurosurgery, Xuanwu Hospital, Capital Medical University, Beijing, China

**Keywords:** *Ascl2*, neural stem cells, miR-26a, in ovo, TNFα

## Abstract

The molecular mediators underlying the effects of inflammation on neural stem cells (NSCs) are not fully characterized. In this study, we identified *Ascl2* as a downstream basic helix-loop-helix (bHLH) transcription factor in NSCs following exposure to TNFα. Under normal conditions, *Ascl2* expression is inhibited at post-transcriptional levels by miR-26a, which targets the 3’ untranslated region (UTR) of *Ascl2*. Upon exposure to TNFα, miR-26a expression is reduced, which leads to up-regulation of *Ascl2*. Overexpression of *Ascl2* promotes neuronal differentiation, reduces proliferation, and increases the level of cleaved CASPASE 3 in NSCs, as observed in the *in vitro* and *in ovo* experiments. *Ascl2* may serve in NSCs as a standby factor that readily responds to TNFα, which is often induced in inflammatory situations. In a chronic inflammatory condition with consistent up-regulation of TNFα, overexpression of *Ascl2* may inhibit neurogenesis as a net result.

Neural stem cells (NSCs) and NSC derivatives possess potentials for treatment of various neurological disorders/diseases, such as Parkinson’s disease, amyotrophic lateral sclerosis (ALS), and spinal cord injury, etc. Autologous vs. allogeneic donor cells/grafts have their pros and cons; and in a foreseeable future, the two would co-exist in medical practices. Our previous study has shown that, allogeneic NSCs can trigger immune recognition when transplanted into the native neurogenic areas in mouse brains, although brain has been considered an immune-privileged organ [1]. The immunological responses mediated mainly through microglia and microglia-secreted cytokines, such as TNFα, exert influences on both the incoming grafts and the endogenous NSCs [1, 2]. Particularly, the effects of TNFα are context-dependent and the net result of TNFα signaling pathway can be either deleterious or beneficial in different models, for example, in allograft transplantation models and in cranial irradiation models [2]. The contribution of inflammatory components in modulation of neurogenesis seems to be complex, and the downstream mediators in acute and chronic inflammation have not been fully characterized.

Achaete-scute complex homolog 2 (*Ascl2*) is a basic helix-loop-helix (bHLH) transcription factor, a mammalian member of the *achaete-scute* family. *Ascl2* expression is relatively high in placenta, Schwann cells, and intestine stem cells, but is normally low or undetectable in other tissues/organs [3-5]. In murine placentas, *Ascl2* is necessary for the specification of spongiotrophoblast cells [6]. In mouse peripheral nervous system, *Ascl2* is expressed in Schwann cells and serves as a negative regulator for Schwann cell proliferation. After sciatic nerve lesion, *Ascl2* level in Schwann cells is down-regulated to promote proliferation [4]. In mouse small intestines, *Ascl2* is considered as an intestine stem cell marker whose expression is critical for maintaining the stemness of intestine stem cells [5]. And ectopic overexpression of *Ascl2* seems to be implicated in colorectal cancer [7-9]. However, in *Drosophila* posterior midgut, *Scute* plays a different role. Transient *Scute* activation instructs intestine stem cells to assume asymmetric divisions, which generates a stem cell and an enteroendocrine progenitor cell. Scute activity then guides each enteroendocrine progenitor cell to divide exactly once before terminally differentiating to a pair of enteroendocrine cells [10]. The seemingly different roles in the above studies suggest that the complex functions of *Ascl2* may be tissue-specific and microenvironment-dependent.

In the current study, we found that *Ascl2* is induced in NSCs by treatment with pro-inflammatory cytokine TNFα, and *Ascl2* affects the proliferation and differentiation of NSCs.

## MATERIALS AND METHODS

### Neural stem cell culture

Mouse NSCs were isolated from postnatal day 0 pups of C57BL/6 background provided by the Jackson Laboratory. Using methods previously described [2], the cerebellum and brain stem were removed from whole brains of neonatal animals, the remnant tissues were enzymatically digested and triturated by pipetting. The resultant cell suspension was centrifuged and re-suspended in growth medium-Neurobasal A supplemented with B-27 without vitamin A, L-glutamax, FGF2 and EGF (ThermoFisher, Waltham, MA, USA), to allow for selective survival of NSCs and formation of neurospheres. When neurospheres grew bigger, the spheres were passaged by treatment with Accutase (ThermoFisher). For monolayer culture, the spheres were seeded in plates coated with poly-D-lysine/laminin to allow for attachment and spreading. An evenly distributed monolayer culture could be achieved after two cell passages.

### Pathway focused array

To examine the transcriptional changes in stem cell-related genes downstream of TNFα treatment in murine NSCs, we performed a pathway focused array (GEArray Express Mouse Neurogenesis and Neural Stem Cell Microarray, EMM-404). NSCs of passage number 7 (P7) were cultured as monolayer in a differentiation medium for 5 h with or without 20 ng/ml TNFα and/or SN50 (an inhibitor that blocks NF-kB to translocate to nucleus). Two hundred and eighty-one genes related to neural stem cells plus *Gapdh* as an internal control gene were analyzed.

### Western Blotting

NSCs were lysed by cold RIPA lysis buffer (Applygen Technologies, Beijing, China), and the protein concentrations were determined by using BCA Protein Assay Reagent (ComWin Biotech, Beijing, China). The lysates were separated by 10% SDS-PAGE and transferred electrophoretically onto polyvinylidene difluoride membranes. After being blocked with 5% non-fat milk in TBST, the membranes were incubated with the primary antibody against ASCL2 (MAB4418, Merk Millipore, Burlington, Massachusetts, USA). After incubation with an HRP-conjugated secondary antibody (ComWin Biotech), the signals were measured by using ECL reagents (Merk Millipore) and visualized by the ChemiDoc MP imaging system. GAPDH (ComWin Biotech) was used as an internal control.

### Quantitative Real-Time PCR (qPCR)

To detect mRNA and miRNA from NSCs, RNA was harvested from cells using the RNEasy kit (Qiagen, Duesseldorf, Germany) or TRIzol reagent (ThermoFisher) according to the previously instruction [11]. Total cDNA was obtained by using PrimeScript™ RT reagent (Takara, Tokyo, Japan). Quantitative RT-PCR was performed using SYBR Premix Ex Taq II (Takara). *Gapdh* was used as an internal control. The miRNA-specific cDNA generation and RT-PCR were performed using Hairpin-it TM miRNAs RT-PCR Quantitation Kit (GenePharma, Shanghai, China), and all the Taqman probes listed in Table S2 were synthesized by GenePharma. Signals were detected using an LC480 Real-Time PCR system (Roche, Basel, Switzerland).

### Cell cycle analysis

Mouse NSCs were seeded on PDL/Laminin-coated plates in medium supplemented with 4 ng/ml polybrene solution with pBMN-*Ascl2*-GFP (retrovirus) or pBMN-GFP (retrovirus) for 24 h. The retroviral vector pBMN-*Ascl2*-GFP was constructed by inserting the mouse *Ascl2* coding sequence (800 bp). After the virus was washed off, the cells were incubated in new medium for 24 h, then dissociated with Accutase, and re-suspended into single cell solution. GFP-positive cells were sorted by using BD FACSAria II (Becton Dickinson, CA, USA). Five hours later, cells were fixed in cold 70% ethanol for 2 h at 4°C. Prior to flow cytometric assay, NSCs were incubated with 100 μg/ml RNase for 30 min and then with 100 ug/ml Propidium Iodide (PI, Sigma-Aldrich) for 15 min. In order to reduce the loss of cells during the process, 1% BSA was added to the washing buffer.

### BrdU labeling

NSCs were infected with pBMN-*Ascl2*-GFP virus. After sorting, cells were seeded in PDL/Laminin-coated coverslips in proliferation medium. Twenty-four hours later, the culture was pulsed with 5 μM BrdU for 1 h, followed by medium change and cultured for another 15 h. Then the cells were fixed in 4% paraformaldehyde (PFA) for 10 min at room temperature. The staining procedure for BrdU has been described in a previously published study [11].

### In ovo electroporation of chicken embryos

As previously described [12, 13], fertilized eggs were incubated for 36 h on one side in a humidified incubator at 37 °C to reach Hamburger-Hamilton (HH) stage10. A pencil was used to mark the top of the egg. At the small end of the egg, approximately 5 ml albumin was removed carefully by using a syringe with 18G needle, and then the hole was sealed with a small piece of tape. The top of the egg was covered with another piece of tape (about 4 x 4 cm). A hole of appropriate size was cut for windowing. Afterwards, 5% Fast Green was injected under the embryo to facilitate visualization of the embryo. Plasmid solution was made by mixing 0.8 μl of 5 % Fast Green and 7.2 μl of pCIG-*Ascl2*-GFP or pCIG-GFP (3 μg/μl) and was injected into the lumen of the neural tube until the dye filled the entire space. For electroporation, the two electrodes spaced 3 mm apart were placed parallel to each side of the embryo. Five times of pulses were conducted at 18 volts at 1 second intervals and lasted for 50 milliseconds. Presence of bubbles close to the negative electrode suggested that the electroporation system had properly worked. Consequently, the eggs were sealed with tape and returned to the incubator. After 24 or 48 h, the embryos with GFP fluorescence were collected for further analysis. A small hole was made at the top of the brain and the embryos were immersed in 4% PFA for 1 h at room temperature, followed by transferring into 30% sucrose solution diluted in 0.1 M phosphate buffer. After sinking to the bottom, the embryos were frozen in OCT embedding solution, cut into slides (20-25 μm) and stored at -80°C.

### Immunohistochemistry

The chicken embryo sections or fixed cells were immunostained as previously described [14]. The following primary antibodies were used in the study: Rat anti-BrdU (1:1000, OBT0030CX, Bio-Rad), mouse anti-NEUN (1:500, MAB377, Millipore), goat anti-DCX (1:500, SC8066, Santa Cruz Biotechnology, Dallas, USA ), rabbit anti-cleaved CASPASE3 (1:500, 9661, Cell Signaling Technology), mouse anti-NESTIN (1:500, 611658, BD Biosciences, San Jose, USA), goat anti-SOX2 (1:1000, SC17320, Santa Cruz Biotechnology), Rabbit anti-P65 (1:500, Santa Cruz Biotechnology). Donkey anti-mouse, rat, goat or rabbit antibodies conjugated to FITC, Cy3, Cy5 or biotin were used at 1:500 dilution (Jackson ImmunoResearch, West Grove, USA).

### Confocal microscopy

A Leica TCS SP5 confocal microscope was used for analysis. Appropriate gain and black level settings were determined on control tissues stained with secondary antibodies alone. Upper and lower thresholds were always set using the range indicator function to minimize data loss through under or over saturation. Upper and lower thresholds were then held constant for all samples when scoring tissues or cells for a given experiment.

### Luciferase reporter assay

Luciferase reporter experiments were performed in the HEK293 or NIH3T3 cells. The wild type and mutant 3′ UTR segment of the *Ascl2* gene was amplified by PCR and inserted to replace the original 3' UTR of the *LuxA* gene in the vector GP-miRGLO (GenePharma). Using Lipofectamine® 2000 Transfection Reagent (ThermoFisher), cells were transfected with 50 ng *Ascl2*-3'UTR or *Ascl2*-3'UTR-MUT (mutation) plasmid and 100 nM miR-26a-5p mimics or control miRNA (NC) (GenePharma). Cells were lysed 24 h post-transfection, and luciferase activity was measured by using dual luciferase assays (E1910, Promega, Wisconsin-Madison, USA) following the manufacturer’s protocol.

### Statistical analysis

All experiments were performed at least three times. The results of one representative experiment were shown in the figures. Data were expressed as means ± SEM and were analyzed by using student t-test or one-way analysis of variance (ANOVA) using Prism 5.0 GraphPad software (GraphPad, San Diego, CA, USA). A p value less than 0.05 was considered statistically significant.

**Figure 1.**
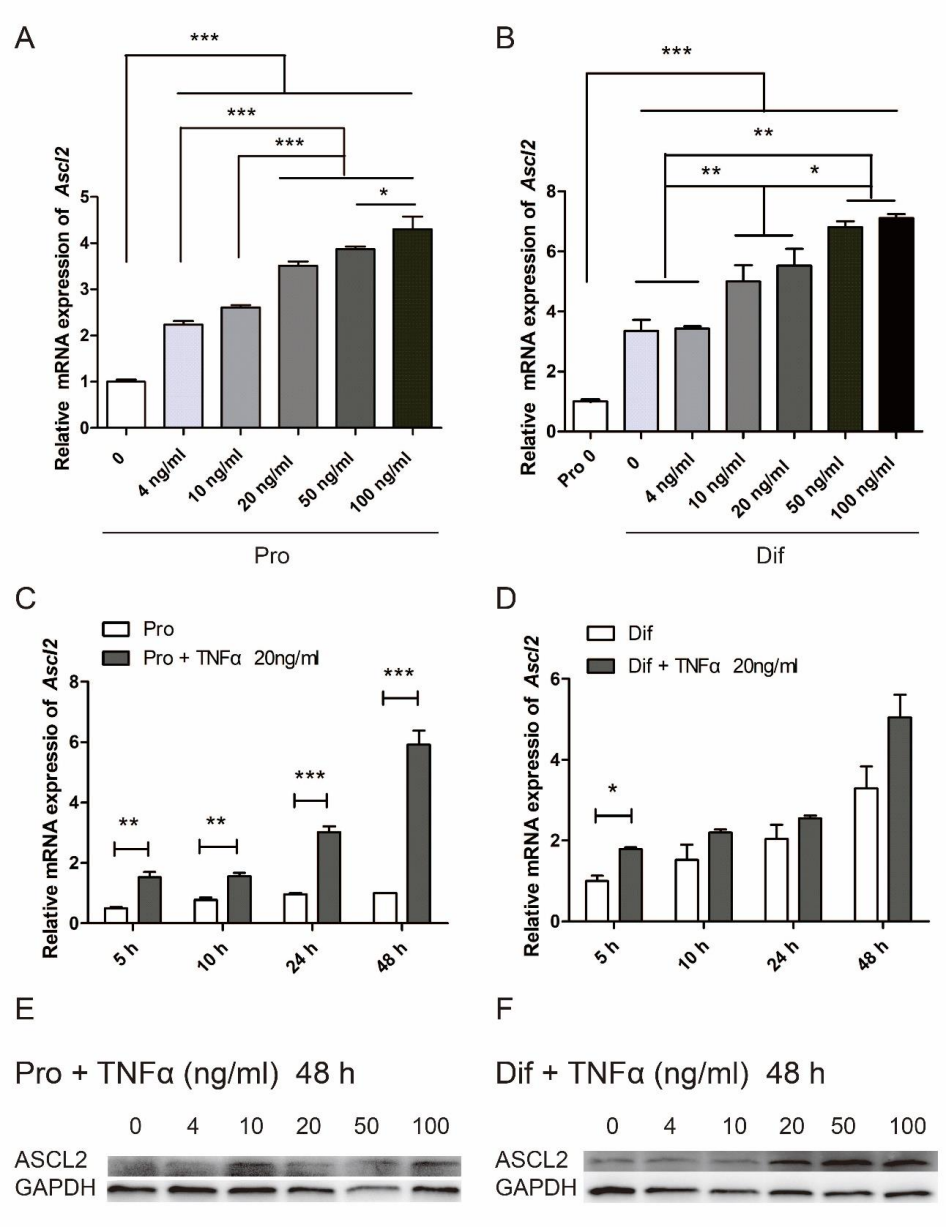
Exposure to TNFα up-regulates *Ascl2* expression in neural stem cells (NSCs). (A-B) Mouse NSCs were treated with different concentrations of TNFα in proliferation (A) and differentiation (B) medium for 48 h, and *Ascl2* mRNA expression levels were examined. The results were normalized to NSCs without TNFα. See also Figure S2. Pro: proliferation, Dif: differentiation. (n=3). (C-D) NSCs were treated with 20 ng/ml TNFα for various lengths of time in proliferation (C) and differentiation (D) medium, and *Ascl2* mRNA expression levels were examined. The results were normalized to NSCs in differentiation medium without TNFα for 5 h. (n=3). (E-F) NSCs were treated with TNFα of different doses for 48 h in proliferation (E) and differentiation (F) medium, and the protein levels of ASCL2 were examined. Data in (A), (B), (C) and (D) are represented as the means ± SEM. *p < 0.05; **p < 0.01; ***p < 0.001 by one-way analysis of variance.

**Figure 2.**
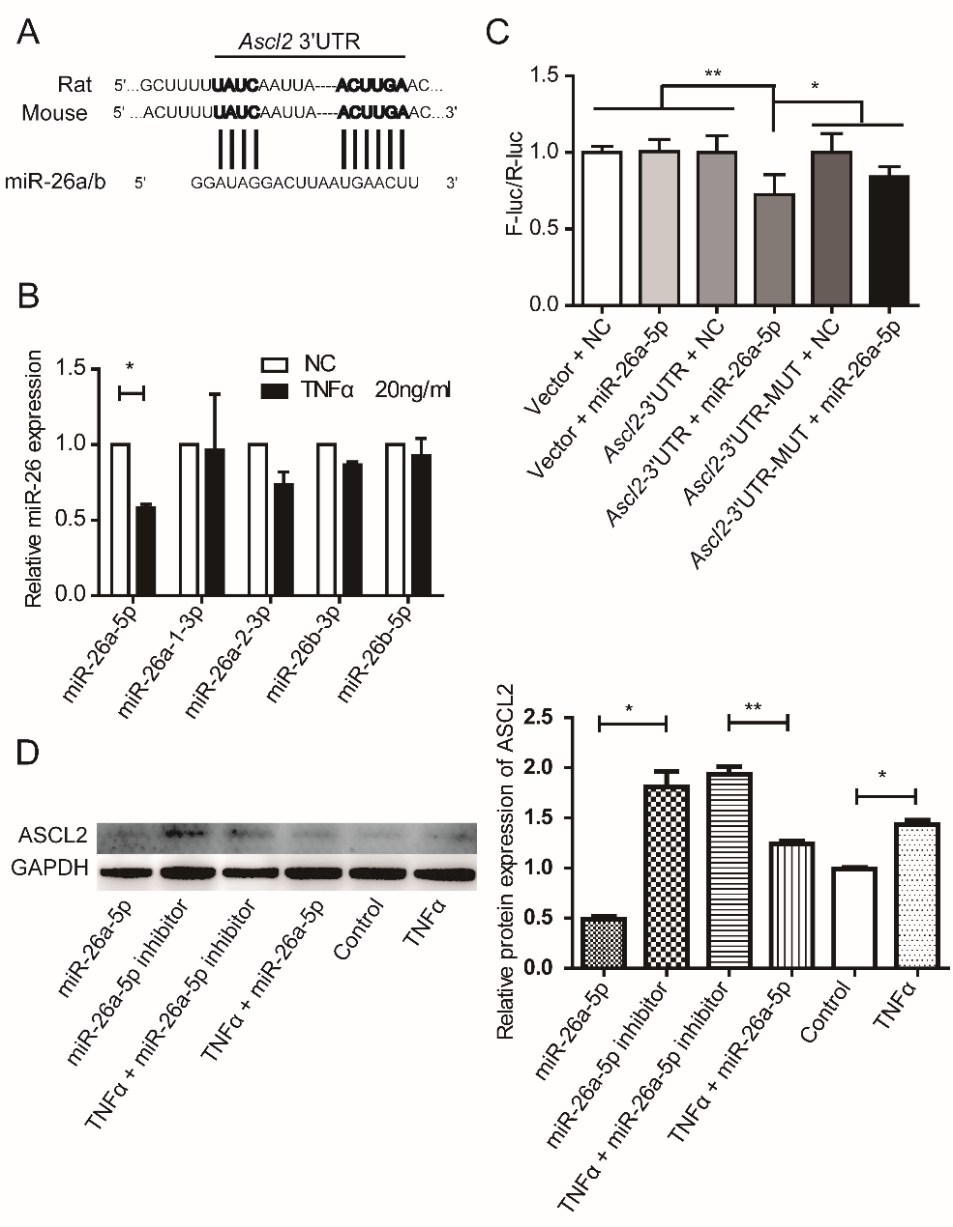
MiR-26a regulates *Ascl2* expression. (A) The alignments of 3’UTR of *Ascl2* and miR-26a/b as predicted by TargetScan analysis. The sequences show a high level of complementarity (indicated by vertical bars) and sequence conservation between mice and rats. (B) Expression levels of different miR-26 family members following TNFα treatment for 5 h in NSCs. The results were normalized to NC. (n=3). (C) NIH3T3 cells were transfected with empty control vectors, *Ascl2* 3’UTR luciferase construct or *Ascl2* 3’UTR mutation luciferase construct together with miR-26a-5p mimics. Forty-eight hours following transfection, cells were collected and lysed. Firefly luciferase activities were examined and normalized to Renilla luciferase activities. (n=6). Similar results were obtained in 293T cell line as shown in Figure S4C. The results were normalized to vector + NC. (D) NSCs were transfected with miR-26a-5p mimics or miR-26a-5p inhibitor with or without TNFα for 48 h, and ASCL2 protein levels were examined. Control means NSCs culture in proliferation medium without TNFα or MicroRNA. (n=3). Data in (A), (C) and (D) are represented as the means ± SEM. *p < 0.05; **p < 0.01; ***p < 0.001 by Student’s t test for comparison (B) and one-way analysis of variance (C) and (D).

## RESULTS

### Exposure to TNFα up-regulates Ascl2 expression in neural stem cells.

Our previous study showed that TNFα differentially regulates NSCs and neuronal precursors through transcriptional and apoptotic pathways, respectively [2]. To examine the transcriptional changes in stem cell-related genes downstream of TNFα treatment in murine NSCs, we performed a pathway focused array (GEArray Express Mouse Neurogenesis and Neural Stem Cell Microarray, EMM-404). NSCs of P7 were cultured as monolayer in a differentiation medium for 5 h with or without 20 ng/ml TNFα and/or SN50 (an inhibitor that blocks translocation of NF-kB to nucleus). Two hundred and eighty-one genes related to neural stem cells plus *Gapdh* as an internal control gene were examined (Supplementary Fig. 1). Among these, 8 genes (*Ascl2, Hes6, P21, Nentrin1, JHDM3A, NeuroD, GFAP, Mash1*) were chosen for confirmation with quantitative PCR (qPCR, the primer information was listed in Table S1); in addition, cells cultured under a proliferation condition (Neurobasal A, L-glutamine, B-27 without vitamin A, 20 ng/ml FGF-2, and 20 ng/ml EGF) for 5 h and a differentiation condition (Neurobasal A, L-glutamine, B-27 without vitamin A) for 72 h with or without TNFα were added for the confirmation test (Supplementary Fig. 2). *Ascl2* expression increased and *Hes6* expression decreased following TNFα treatment, which was partially reversed by SN50 treatment under the 5 h differentiation condition (Supplementary Fig. 2A). Under conditions of differentiation for 72 h or proliferation for 5 h, *Ascl2* expression also consistently increased and *Hes6* decreased with TNFα treatment (Supplementary Fig. 2B and 2C). TNFα treatment up-regulated expression of *p21* and *Netrin1* (Supplementary Fig. 2). The role of *Ascl2* in neural stem cells has largely been unexplored, and therefore we focused this study on this particular gene.

**Figure 3.**
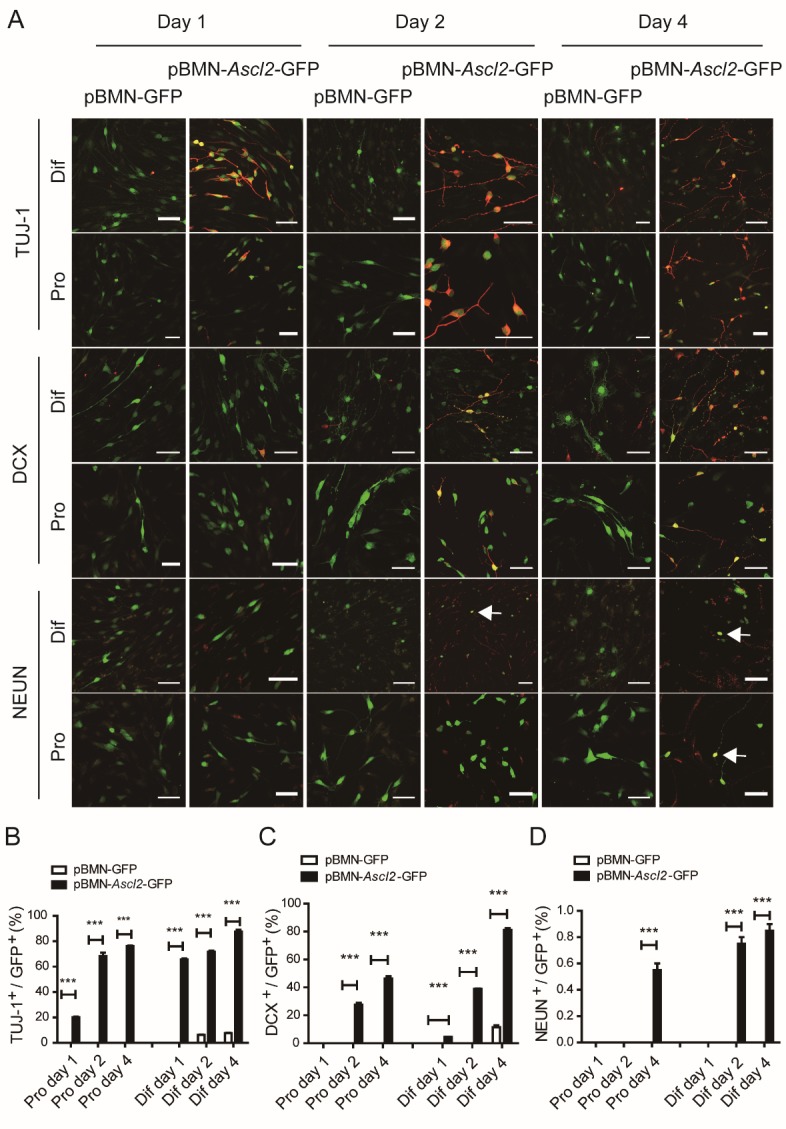
*Ascl2* affects the differentiation of NSCs *in vitro.* (A) Monolayer murine NSCs were infected with retrovirus encoding pBMN-*Ascl2*-GFP or pBMN-GFP, which were then subjected to a differentiation or proliferation condition for 1, 2 and 4 days. Early neuronal markers TUJ-1 and DCX, and mature neuron marker NEUN were stained. Pro, proliferation; Dif, differentiation. Scale bars, 50 μm. (B-D) The proportions of TUJ-1-, DCX- and NEUN-positive cells among GFP^+^ cells were scored. (n=10-18). Data in (B-D) are represented as the means ± SEM. *p < 0.05; **p < 0.01; ***p < 0.001 by Student’s t test for comparison.

**Figure 4.**
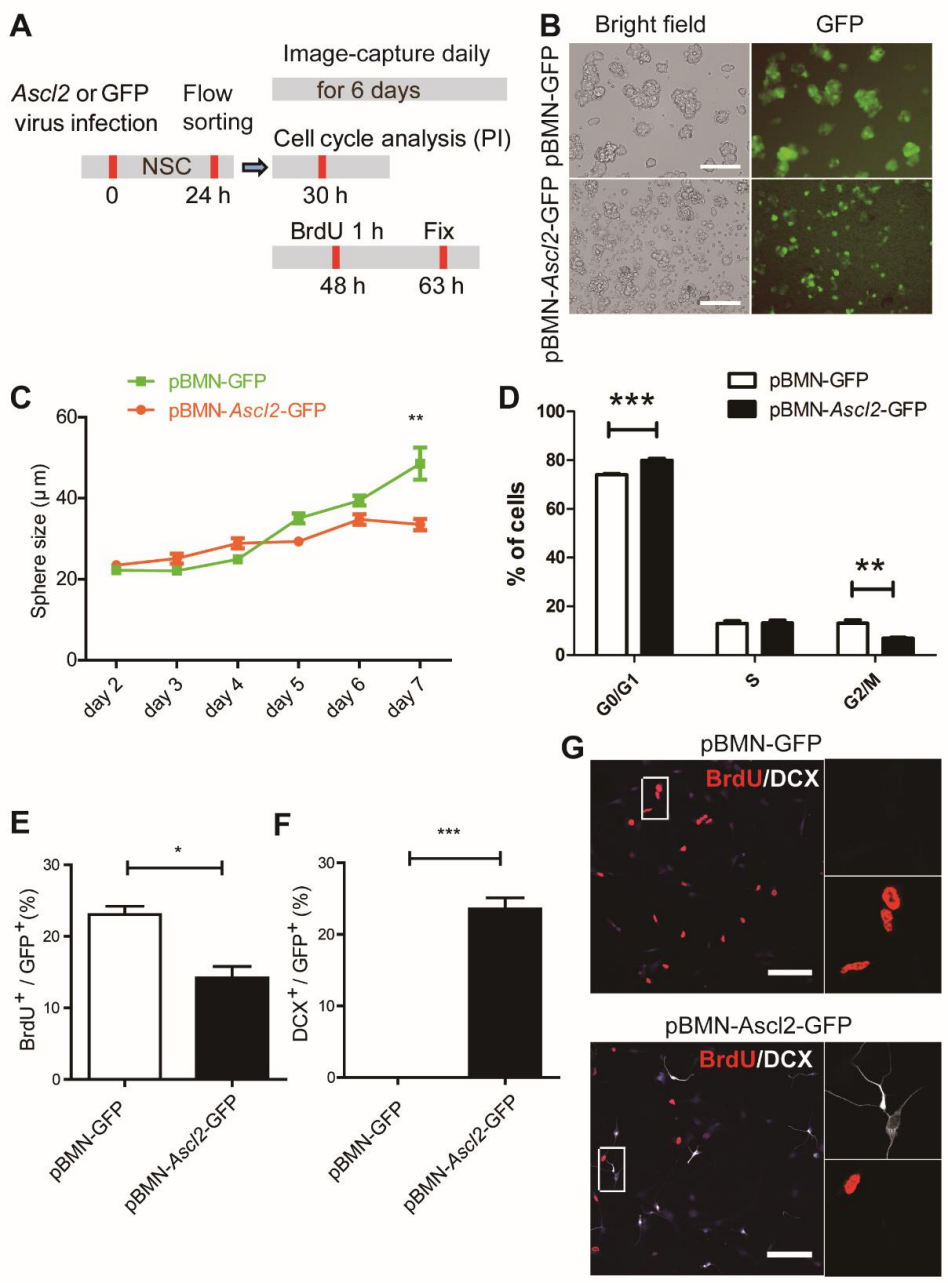
The impact of *Ascl2* overexpression on the proliferation of NSCs. (A) Schematic representation of the experimental procedures. (B) The flow cytometer-sorted single cells were cultured in a proliferation medium to test the ability to form neurospheres. Representative pictures 5 days after flow cytometer sorting were shown. (C) The sphere sizes were measured from day 2 through day 7 after flow cytometer sorting. (n=17-47). (D) Cell cycle progression in proliferation condition was analyzed by PI staining and flow cytometry. (n=3). (E-F) The proliferative capacity was tested by BrdU pulsing for 1 h and immunostaining 14 h later. The proportions of BrdU^+^ and DCX^+^ cells among GFP^+^ cells were scored respectively. (n=3). (G) Co-labeling of BrdU with DCX. Scale bars, 100 μm. Data in (C), (D), (E) and (F) are represented as the means ± SEM. *p < 0.05; **p < 0.01; ***p < 0.001 by Student’s t test for comparison.

**Figure 5.**
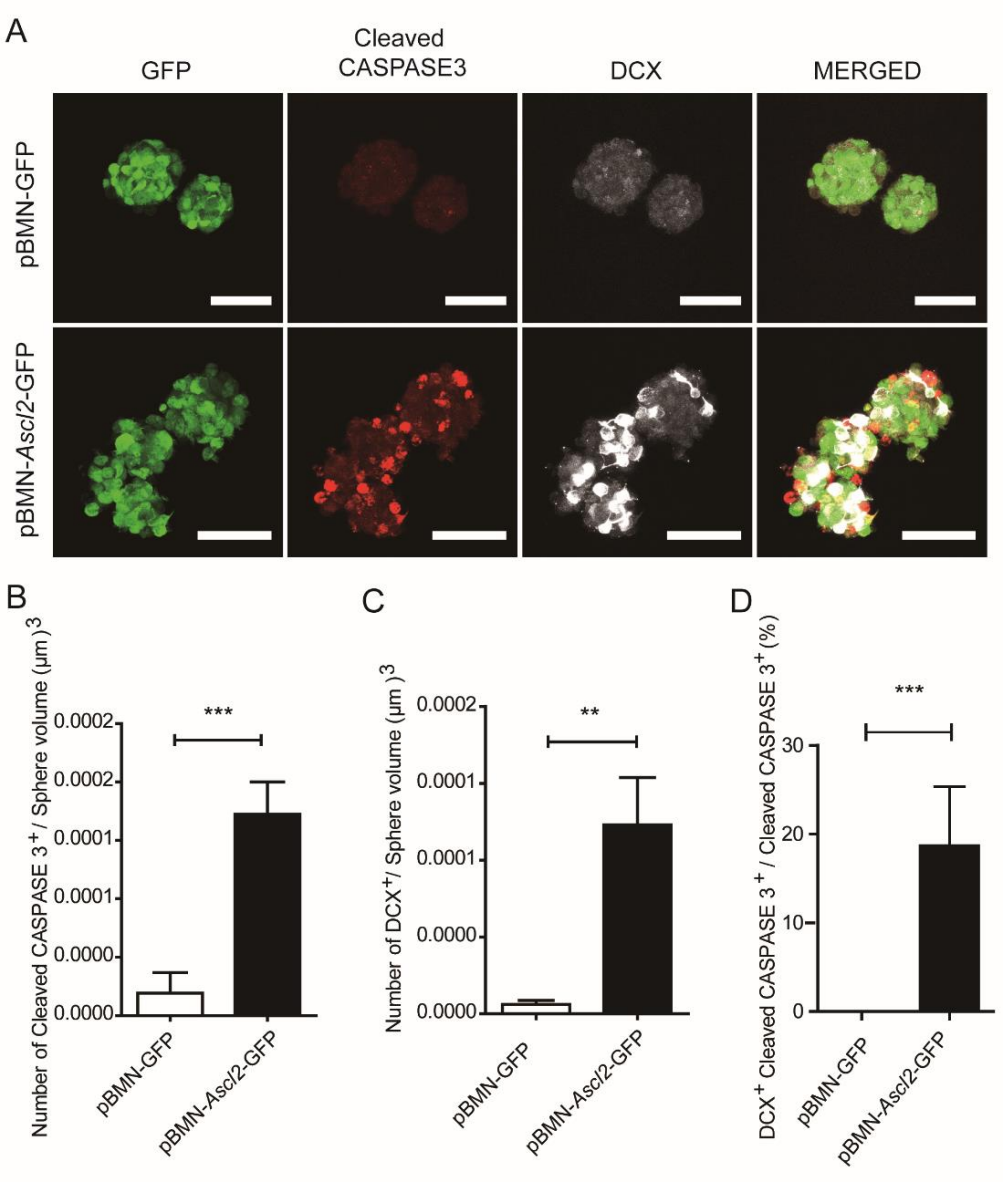
Overexpression of *Ascl2* induces expression of cleaved CASPASE 3 in neurospheres. (A) NSCs infected with retrovirus encoding *Ascl2* and GFP or GFP only were sorted by using flow cytometry, 24 h later, the spheres were immunostained for cleaved CASPASE 3 and DCX. Scale bars, 50 μm. (B-C) The cells positive for cleaved CASPASE 3 and DCX were quantified respectively. (n=9-11). (D) The proportion of cleaved CASPASE 3-positive cells co-expressing DCX was scored. (n=9-11). Data in (B), (C) and (D) are represented as the means ± SEM. **p < 0.01; ***p < 0.001 by Student’s t test for comparison.

We first investigated *Ascl2* expression under more thorough conditions that included different doses of TNFα (0, 4, 10, 20, 50, and 100 ng/ml) for 5, 10, 24, and 48 h either in proliferation or differentiation medium (Fig. 1A and B). Under the proliferation condition, NSCs expressed NSC-specific markers NESTIN and SOX2 (Supplementary Fig. 3A). When NSCs were differentiated for 7 days, mature NEUN+ neurons started to appear, and the proportion of mature neurons continued to increase over time (Supplementary Fig. 3B), proving that the differentiation system worked. Samples from various conditions were run in parallel so that values could be compared between conditions. In general, *Ascl2* mRNA levels were increased with higher concentrations of TNFα treatment for 48 h, both in proliferation and differentiation medium (Fig. 1A and B). Interestingly, *Ascl2* expression was higher in differentiation vs. proliferation condition (Fig. 1B). Following treatment of TNFα (20 ng/ml) for various lengths of time, *Ascl2* levels were increased, with a longer period of exposure to TNFα generally corresponding to a higher level of *Ascl2* (Fig. 1C and D). Along the course of differentiation, *Ascl2* expression was up-regulated over time (Fig. 1D), indicating a possible role in NSC differentiation. Because *Ascl2* expression increased in the differentiation medium but not in the proliferation medium (without TNFα treatment), TNFα-induced up-regulation of *Ascl2* was more marked in proliferation medium vs. in differentiation medium (Fig. 1C and D). The protein levels of ASCL2 as examined by using Western blot were enhanced by TNFα treatment in a dose-dependent manner (Fig. 1E and F).

### Mir-26a may mediate regulation of Ascl2.

Two receptors of TNFα exist - TNFR1 and TNFR2, which play different, sometimes even opposing roles in a context-dependent manner [2]. We used NSCs isolated from TNFR1^-/-^ and TNFR2^-/-^ mice to determine whether TNFR1 or TNFR2 was involved in regulation of *Ascl2* by TNFα. In TNFR1^-/-^ NSCs, the effect of TNFα on *Ascl2* was abolished, suggesting that TNFR1, but not TNFR2, mediates the effect (Supplementary Fig. 4A).

Next, we tested whether TNFα regulates *Ascl2* through transcriptional activation. The promoter region of *Ascl2* from -968 to +444 bp relative to the transcription start site (TSS) was cloned into pGL3 vector to drive the downstream luciferase gene expression. A web-based software (LASAGNA-Search 2.0) predicted a possible NF-kB binding site with a score of 79.7 at -310 bp relative to TSS (Supplementary Fig. 5). Murine NSCs cultured as monolayer were transfected with pGL3-*Ascl2* promoter together with Renilla control vectors. The next day the cells were treated with TNFα and/or SN50 for 5 or 24 h. Twenty-four hours after treatment, the cells were lysed for dual luciferase assay. The results showed that TNFα pathway did not regulate *Ascl2* by transcriptional activation of the tested promotor region (Supplementary Fig. 4B).

By using the software TargetScan, it was predicted that miR-26 may target the 3’ untranslated region (UTR) of *Ascl2* (Fig. 2A). Wei et al. showed that NF-kB pathway down-regulates miR-26a in cardiac fibrosis [15]. NF-kB pathway is activated in NSCs following TNFα treatment [2], which was also confirmed in the current study (Supplementary Fig. 3C). We therefore hypothesized that TNFα might regulate *Ascl2* expression through miR-26a. To test this hypothesis, we first examined whether TNFα treatment could reduce miR-26a expression in NSCs. NSCs were cultured as monolayer and treated with or without TNFα for 5 h, and different miR-26 family members (mmu-miR-26a-5p, mmu-miR-26a-1-3p, mmu-miR-26a-2-3p, mmu-miR-26b-5p, mmu-miR-26b-3p) were examined (Fig. 2B). Among them, only miR-26a-5p was significantly down-regulated. Next, we cloned the 3’UTR of *Ascl2* to replace the 3’UTR of luciferase gene in a pGL3-Luc vector, and tested whether miR-26a-5p could affect luciferase expression in a NIH3T3 system (Fig. 2C) and 293T (Supplementary Fig. 4C). In both systems, delivery of miR-26a-5p into cells by using a Lipofectamine® RNAiMAX could significantly reduce luciferase activity (normalized by Renilla in a dual luciferase system), suggesting that miR-26a might directly target the 3’UTR of *Ascl2* and down-regulate its expression. To confirm this possibility, we delivered miR-26a mimic (5’UUCAAGUAAUCCAGG AUAGGCU3’) and its inhibitor (5’AGCCUAUCC UGGAUUACUUGAA3’) into NSCs with or without TNFα, and then examined ASCL2 protein expression by using Western blot. MiR-26a-5p but not its inhibitor significantly decreased the protein levels of *Ascl2* following TNFα treatment (Fig. 2D).

### *Ascl2 affects the differentiation and proliferation of NSCs in vitro*.

To investigate the role of *Ascl2* in NSCs, retrovirus encoding *Ascl2* and GFP (pBMN-*Ascl2*-GFP) or GFP only (pBMN-GFP) as controls were used to infect monolayer murine NSCs, which were then subjected to a differentiation or proliferation scheme. The infection rate was about 15%, and the green cells were selected for analysis (Fig. 3A). Early neuronal markers TUJ-1 and DCX, and mature neuron maker NEUN were stained and scored among the GFP^+^ cells. In the proliferation condition, few TUJ-1^+^ or DCX^+^ neuronal precursors were observed in the control group infected with GFP virus only at any time points tested (Fig. 3A). In contrast, infected cells overexpressing *Ascl2* showed a premature neuronal differentiation even in the proliferation condition. On day 1, there were already 20.1 ± 0.37 % TUJ-1^+^ cells among the green cells; and on day 4, about 74.8 ± 1.26 % TUJ-1^+^ cells and 45.8 ± 1.38 % DCX^+^ cells were detected in the proliferation condition (Fig. 3A-C). In the differentiation condition, the percentages of TUJ-1^+^ and DCX^+^ cells in control group gradually increased and reached about 10% on day 4 (Fig. 3A-C). In the *Ascl2*-overexpressing group, two days of differentiation already led to more than 70% of TUJ-1^+^ and around 40% of DCX^+^ cells; on day 4, the proportion increased to more than 80% for TUJ-1^+^ and DCX^+^ cells (Fig. 3B and C), > 8-fold higher than that in control group. As to mature neuronal marker NEUN, no positive cells were observed in control groups in any of the conditions tested. It normally took 7 days for the NEUN^+^ mature neurons to appear in the control group under the current experiment setting (Supplementary Fig. 3B). But in the *Ascl2*-overexpressing group, there were about 0.6% and 0.9% NEUN^+^ cells detected among the green cells on day 4 of proliferation and differentiation, respectively (Fig. 3D). The results suggested that *Ascl2* expression resulted in a premature neuronal differentiation of NSCs.

Next, we examined the impact of *Ascl2* on the proliferation aspect of NSCs. NSCs infected with retrovirus encoding *Ascl2* and GFP or GFP only were sorted by using flow cytometer, and the GFP^+^ cells were subjected to sphere culture, cell cycle analysis, and BrdU pulse experiments (Fig. 4A). The single cells sorted by flow cytometer were cultured in a proliferation medium to test the ability to form neurospheres (Fig. 4B and C). On day 5, *Ascl2*-expressing spheres looked ragged on surface, compared with control cells (Fig. 4B). The sphere sizes were quantified from day 2 through day 7, and *Ascl2* spheres were significantly reduced in size from day 7 (Fig. 4C). The cell cycle progression in the proliferation condition was analyzed by PI staining of the nuclear content. *Ascl2*-expressing NSCs showed a prolonged G0/G1 phase and reduced G2/M phase (Fig. 4D). The proliferative capacity was also tested by BrdU pulsing for 1 h and immunostaining 14 h later. The proportion of BrdU^+^ cells was significantly lower in *Ascl2*-expressing group vs. control group (Fig. 4E and G), in agreement with an enhanced percentage of DCX^+^ cells (Fig. 4F and G).

**Figure 6.**
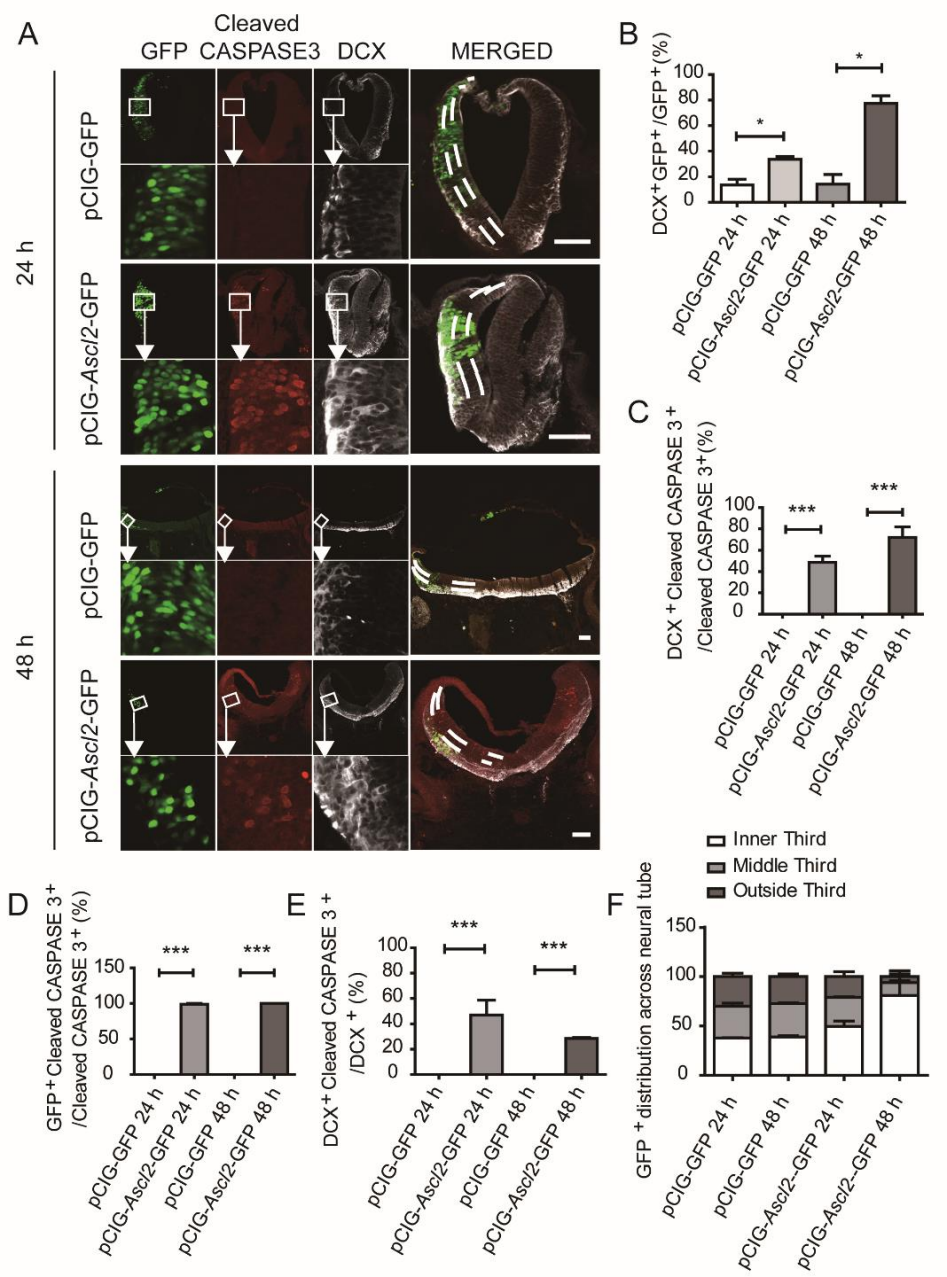
Overexpression of *Ascl2* induces expression of cleaved CASPASE 3 *in ovo.* (A) PCIG-*Ascl2*-GFP vectors and the control vectors pCIG-GFP were electroporated into chicken embryo neural tubes, 24 h and 48 h later, the chicken embryos were fixed and sliced for staining of cleaved CASPASE 3 and DCX. Scale bars, 200 μm. (B) The proportions of GFP^+^ cells co-expressing DCX were scored 24 h and 48 h after electroporation. (n=6). (C) The proportions of cleaved CASPASE3-positive cells co-expressing DCX were scored 24 h and 48 h after electroporation. (n=6). (D) The proportions of cleaved CASPASE3-positive cells that were GFP-positive were scored 24 h and 48 h after electroporation. (n=6). (E) The proportions of DCX-positive cells that co-expressed cleaved CASPASE3 were scored 24 h and 48 h after electroporation. (n=6). (F) Distribution of GFP^+^ cells in the neural tubes. (n=6). Data in (B), (C), (D) and (E) are represented as the means ± SEM. *p < 0.05; **p < 0.01; ***p < 0.001.

**Figure 7.**
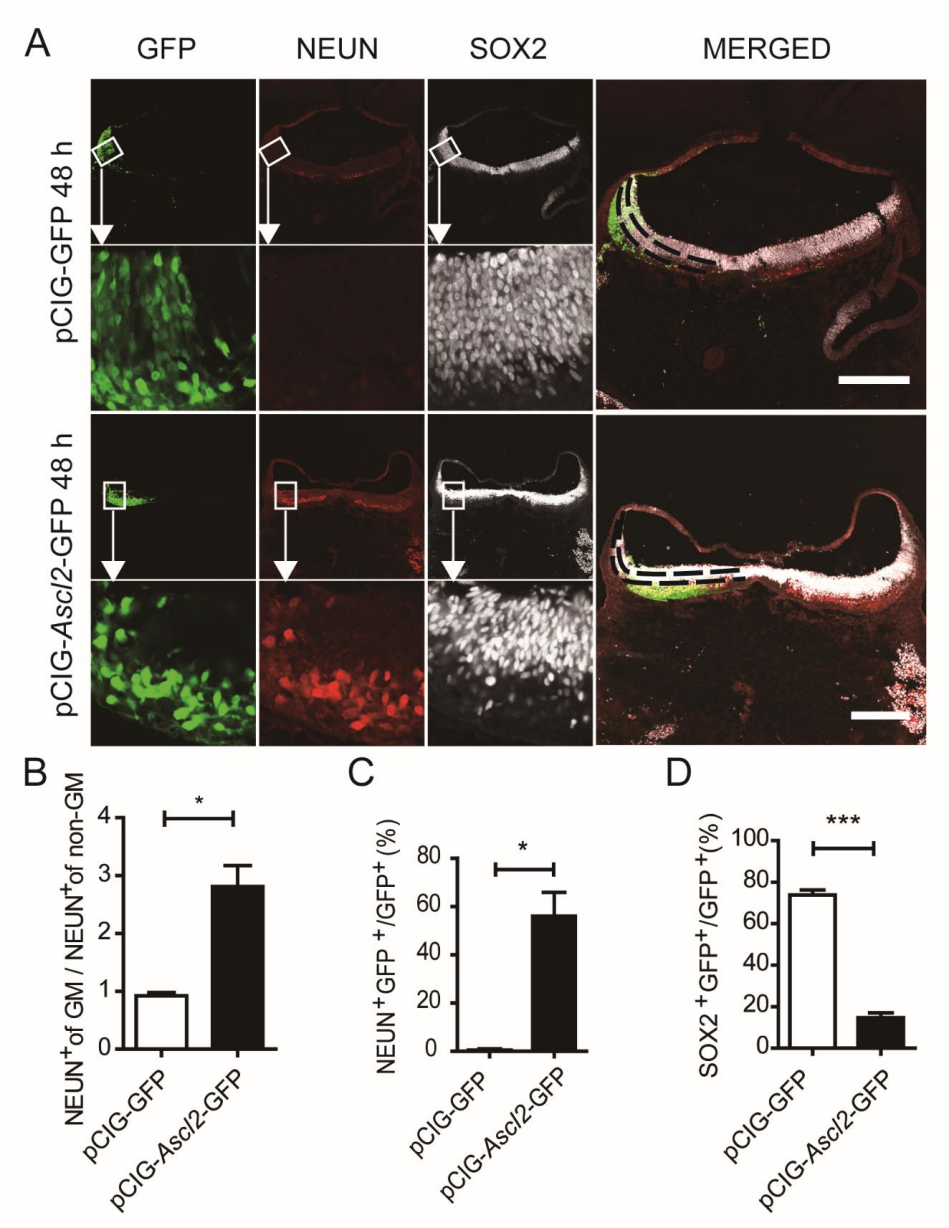
The impact of *Ascl2* overexpression on differentiation *in ov*o. Related to Figure S6. (A) Expression of neural stem cell marker SOX2 and mature neuron marker NEUN at 48 h post-electroporation. (B) Rate of NEUN-positive cells of genetically modified (GM) side over non-genetically modified (non-GM) side 48 h post-electroporation. (n=6). (C) The proportion of NEUN- and GFP-double positive cells in GFP^+^ cells 48 h post-electroporation. (n=6). (D) The proportion of SOX2- and GFP-double positive cells in GFP^+^ cells 48 h post-electroporation. (n=6). Data in (B), (C) and (D) are represented as the means ± SEM. *p < 0.05; **p < 0.01; ***p < 0.001 by Student’s t test for comparison.

We also examined the expression of cleaved CASPASE 3 with or without exogenous *Ascl2* expression (Fig. 5). *Ascl2*-expressing NSCs cultured in a proliferation medium showed a much higher proportion of cleaved CASPASE 3^+^ cells, and among them, about 18.7 % were DCX-positive (Fig. 5). Fernando et al. has shown that expression of active CASPASE 3 can be a conserved feature of neuronal differentiation in non-apoptotic cells [16]. The higher expression of cleaved CASPASE 3 may be a result from *Ascl2*-induced neuronal differentiation *per se*, and/or from an increased level of apoptosis. Further investigation is needed to address this issue.

The in vitro data showed that *Ascl2*-expression led to a premature neuronal differentiation, reduced proliferation, and increased expression of cleaved CASPASE 3 in NSCs.

### In ovo electroporation of Ascl2 leads to early neuronal differentiation.

Next, we examined the function of *Ascl2 in ovo*. pCIG-*Ascl2*-GFP plasmid and control construct pCIG-GFP were injected into neural tubes of Hamburger-Hamilton (HH) stage 10 chicken embryos, followed by application of electric field for 50 milliseconds at 18 volts. The negatively charged vectors migrated toward the positively charged electrode positioned at one side of the developing neural tube, and the other side was used as an internal control (Supplementary Fig. 6A). Twenty-four or 48 h later, the chicken embryos were fixed and sliced for staining to characterize neuronal differentiation.

In the developing neural tubes, neural stem cells are localized more at the lumen side and neuronal precursors and mature neurons more at the outside edges (Figs. 6 and 7). The horizontal sections of neural tubes were arbitrarily divided into 3 equal parts for analysis on the vector side. At 24 h post-electroporation, the GFP^+^ cells were distributed across the 3 arbitrary sections by 37.9 ± 0.3 % (outside third), 32.2 ± 2.2 % (middle third), and 30.0 ± 2.4 % (inner third), in the control vector group. The distribution of GFP^+^ cells across the 3 arbitrary sections were 49.5 ± 3.9 % (outside third), 29.5 ± 0.4 % (middle third), and 21.0 ± 3.6 % (inner third), in the pCIG-*Ascl2* group (Fig. 6A and F); at 48 h, the GFP^+^ cells were distributed by 38.9 ± 1.0 % (outer third), 33.6 ± 0.8 % (middle third), and 27.5 ± 1.8% (inner third), in the control vector group. The GFP^+^ cells were distributed by 80.8 ± 10.5 % (outer third), 13.1 ± 6.2 % (middle third), and 6.1 ± 4.3 % (inner third), in the pCIG-*Ascl2* group (Fig. 6A and F). From 24 to 48 h, the proportion of DCX^+^ cells among the GFP^+^ cells changed from 13.6 ± 3.1 % to 14.1 ± 5.4 % in the control vector group, and the proportion increased from 33.7 ± 1.6 % to 77.4 ± 4.2 % in the pCIG-*Ascl2* group (Fig. 6A and B). The DCX^+^ cells were mostly observed in the outer third section, suggesting that neural stem cells commit to a neuronal fate during or after migration towards the outside edge of neural tubes. At both 24 and 48 h, pCIG-*Ascl2* groups showed markedly higher proportions of DCX^+^ neuronal precursor cells, compared with those of pCIG-GFP control groups (Fig. 6B), suggesting that *Ascl2* had promoted neuronal differentiation of NSCs *in ovo*.

In control vector groups, either at 24 or 48 h post-electroporation, almost no cleaved CASPASE 3^+^ cells were detected; in contrast, CASPASE 3^+^ cells were observed in pCIG-*Ascl2* groups (Fig. 6A, C, D and E). Almost all the CASPASE 3^+^ cells detected were *Ascl2*-transfected cells (Fig. 6A and D). About 48.5 % and 72.1 % of the CASPASE 3^+^ cells were DCX^+^ (DCX^+^/Casp3^+^) at 24 and 48 h, respectively (Fig. 6A and C). Among the DCX^+^ cells, about 47.1 % and 24.3% were positive for cleaved CASPASE 3 at 24 and 48 h, respectively (Fig. 6A and E).

We also examined neural stem cell marker SOX2 and mature neuron marker NEUN at 48 h post-electroporation (Fig. 7A). Compared to the opposite side of the neural tube without vector transfection, the number of NEUN^+^ neurons was 2.8 ± 0.16 fold higher in the pCIG-*Ascl2*-GFP vector side (Fig. 7B). The proportion of NEUN^+^ in the electroporated GFP^+^ cells were 56.1 ± 10.0 % in the *Ascl2* group vs. 0.5 ± 0.4 % in the control vector group (Fig. 7C). Among the GFP^+^ electroporated cells, 14.7 ± 2.3 % were SOX2^+^ in *Ascl2* group vs. 73.8 ± 1.7% in control vector group (Fig. 7D); similar results were obtained about the proportion of another mature neuron marker MAP2 as shown in Figure S6B, C and D.

The above data suggest that *Ascl2* over-expression can shrink the pool of neural stem cells by inducing premature neuronal differentiation.

## DISCUSSION

In this study, we found that *Ascl2* expression is induced in NSCs following treatment with pro-inflammatory cytokine TNFα; up-regulation of *Ascl2* may be mediated by miR-26a, which targets the 3’ untranslated region of *Ascl2* mRNA and can be down-regulated by TNFα treatment. Overexpression of *Ascl2* inhibits the proliferation, and promotes neuronal differentiation of NSCs, as observed in the *in vitro* and *in ovo* experiments.

The way NSCs respond to inflammation may be context-dependent [1, 2]. Acutely activated microglia, or their conditioned medium, reduces neural progenitor cell survival and prevented neuronal differentiation; whereas chronically activated microglia are permissive to neuronal differentiation [17]. In a context of intracerebral transplantation of allogeneic NSCs in mice, allogeneity-induced inflammatory responses reduce neurogenesis [1]. One of the key pro-inflammatory cytokines in the CNS is TNFα. We have shown that TNFα treatment mainly induces apoptotic pathway in neuronal precursor cells and activates NF-kB pathway in NSCs [2]; yet the detailed molecular mechanisms underlying the effects of TNFα on NSCs at the transcriptional level have not been fully depicted. Through a focused pathway array, we identified *Ascl2* as a downstream transcription factor that was up-regulated in murine NSCs following TNFα treatment. *Ascl2* expression is normally very low or at an undetectable level in tissues/cells others than placenta, Schwann cells, and intestine. Upon exposure to TNFα, the level of *Ascl2* rapidly increased, suggesting that *Ascl2* may be an instantaneous mediator in NSCs in response to TNFα, a cytokine often induced by inflammatory challenges. When NSCs were infected with lentivirus encoding control vectors, *Ascl2* levels also increased (Supplementary Fig. 4D). Virus infection represents a kind of cellular stress. It is possible that cellular stressors, such as inflammation and virus infection, might lead to up-regulation of acute responders like *Ascl2*, and trigger an attempt for NSCs to deal with the exogenous changes/challenges. *Ascl2*-induced neuronal differentiation may reflect such an abortive attempt for NSCs to repair CNS damage by producing more neurons. In Drosophila, transient activity of *Scute* induces intestine stem cells to produce a pair of enteroendocrine cells [10], in agreement with what we found in the current study that *Ascl2* participates in stem cell differentiation.

In many neurological disorders/indications, acute inflammatory responses are often considered to play a collectively positive role, facilitating the removal of debris and regeneration of the CNS. However, chronic inflammation is generally regarded as aberrant regulation of the immune system that contributes to certain pathological features of the diseases. In the current study, overexpression of *Ascl2* in the *in vitro* and *in ovo* experiments may mimic the chronic/long term inflammatory condition. In these experimental settings, *Ascl2* inhibits NSC proliferation, and induces premature neuronal differentiation. The net results are the reduction of neural stem cell pool and decrease in neurogenesis. Whether *Ascl2* is the major mediator in conditions where chronic inflammation inhibits neurogenesis requires further investigation.

Interestingly, *Ascl2* seems to play different, even seemingly opposing roles in different tissues and species. In mouse intestine, *Ascl2* helps to maintain the stem cell signature of intestine stem cells; in mouse placenta, *Ascl2* participates in the differentiation to spongiotrophoblast cells. In Drosophila midgut, *scute* instructs the asymmetric division of intestine stem cells, as well as induces exactly one-time proliferation of enteroendocrine progenitor cells and generation of one pair of enteroendocrine cells. In mouse intestine stem cells, *Ascl2* is the direct target of Wnt signaling; whereas in mouse epidermis, *Ascl2* is regulated by Notch signaling pathway. In Drosophila gut, *Ascl2* forms a self-activating regulatory loop. The context-dependent function/ regulation of *Ascl2* indicates that *Ascl2* may work with different co-factors under different conditions. *Ascl2* can form dimers with nuclear ß-cateninand TCFs [18, 19]. Binding of *Ascl2* to E-box (CANNTG) can be antagonized by another transcription factor HAND1 [20]. In NSCs, whether and how *Ascl2* interacts with other co-factors is still unclear and warrants further study.

In short, *Ascl2* may serve in NSCs as a standby factor in normal conditions. Upon TNFα exposure, *Ascl2* in NSCs readily responds and promotes neuronal differentiation of NSCs. Overexpression of *Ascl2* also affects the proliferation of NSCs. In chronic inflammation in which TNFα is consistently up-regulated, sustained expression of *Ascl2* may inhibit neurogenesis as a net consequence.

## Supplementary Materials

The Supplemenantry data can be found online at: www.aginganddisease.org/EN/10.14336/AD.2018.1028.


