# Supplementary material for: TNFα-induced Up-regulation of Ascl2 Affects the Differentiation and Proliferation of Neural Stem Cells
Source: Aging Dis. 2019 Dec 1;10(6):1207–20. doi: 10.14336/AD.2018.1028 (PMC6844591; doi:10.14336/AD.2018.1028)
Supplement: Supplementary file 1 [file AD-10-6-1207-s.pdf]

SUPPLEMENTARY DATA

TNFα-induced Up-regulation of *Ascl2* Affects the Differentiation and Proliferation of Neural Stem Cells

Zhongfeng Liu[1,2,3], Xuan Wang[1,2,3], Kewen Jiang[4], Xunming Ji[5], Y. Alex Zhang[1,*], Zhiguo Chen[1,2,3,*]

[1]Cell Therapy Center, Beijing Institute of Geriatrics, Xuanwu Hospital, Capital Medical University, and Key Laboratory of Neurodegeneration, Ministry of Education, Beijing, China
[2]Center of Neural Injury and Repair, Beijing Institute for Brain Disorders, Beijing, China
[3]Center of Parkinson's Disease, Beijing Institute for Brain Disorders, Beijing, China
[4]Department of Neurology, the Children's Hospital School of Medicine, Zhejiang University, Hangzhou, China
[5]Department of Neurosurgery, Xuanwu Hospital, Capital Medical University, Beijing, China

© 2018. Liu Z et al. Published online at http://www.aginganddisease.org/EN/10.14336/AD.2018.1028

SUPPLEMENTARY DATA

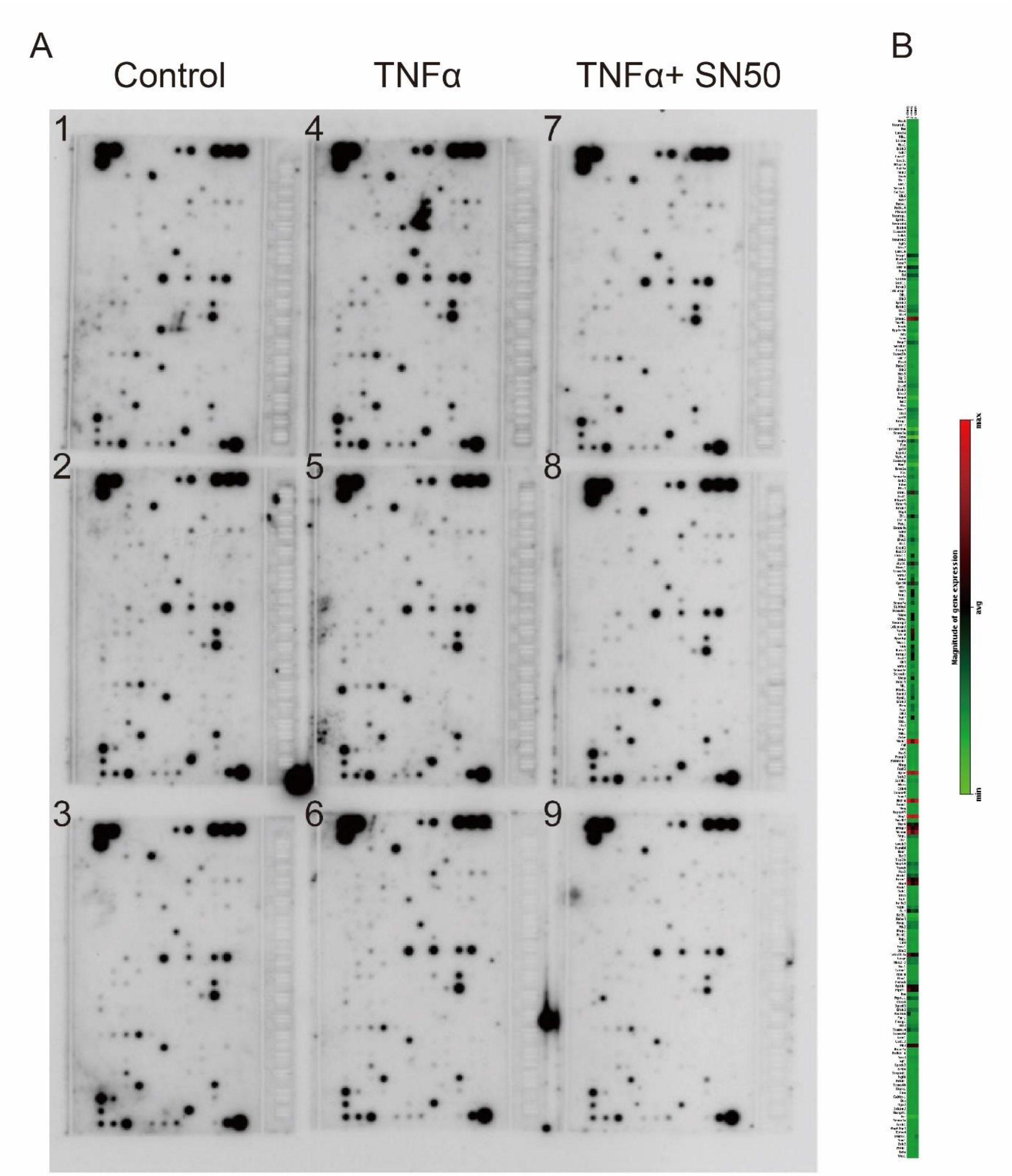

Supplementary Figure 1. Transcriptional changes in stem cell-related genes downstream of TNFα treatment in murine NSCs cultured in a differentiation condition. Related to Figure 1. (A) NSCs of passage number 7 were cultured as monolayer in a differentiation medium. 1, 2, and 3 refer to control group without TNFα treatment; 4, 5, and 6 refer to NSCs treated with 20 ng/ml TNFα for 5 h; 7, 8, and 9 refer to NSCs treated with 20 ng/ml TNFα and 50ug/ml SN50 for 5 h. (n=3). **(B)** Heatmap shows expression of 281 genes related to neural stem cells plus internal control *Gapdh* in the three groups.

© 2018. Liu Z et al. Published online at http://www.aginganddisease.org/EN/10.14336/AD.2018.1028

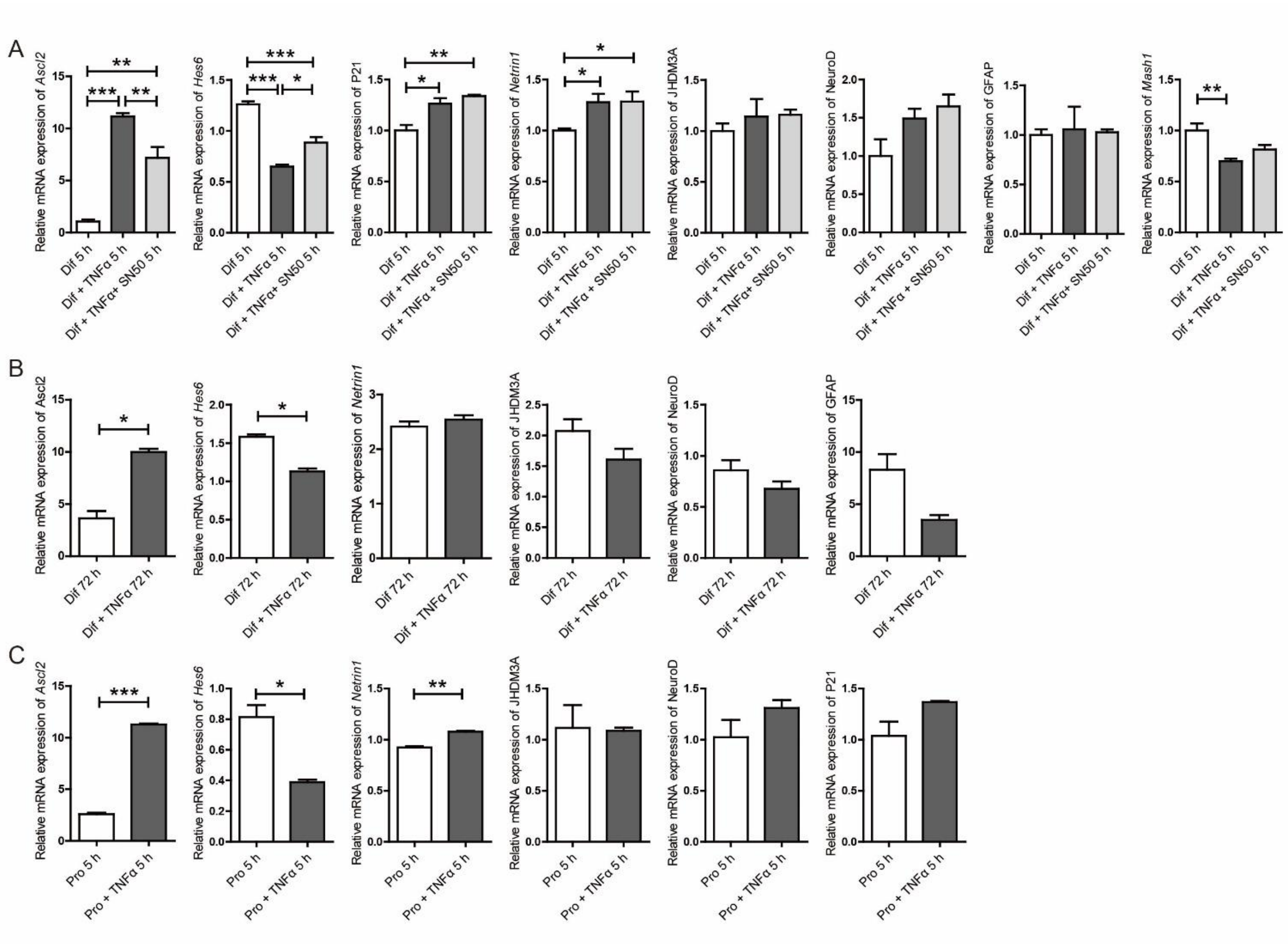

Supplementary Figure 2. qPCR quantification of the mRNA expression levels for *Ascl2, Hes6, P21, Netrin1, JHDM3A, NeuroD, GFAP, and Mash1* under different conditions. **(A)** Differentiation condition for 5 h with or without TNFα and SN50. (n=3). **(B)** Differentiation condition for 72 h with or without TNFα. (n=3). **(C)** Proliferation condition for 5 h with or without TNFα. (n=3).

© 2018. Liu Z et al. Published online at http://www.aginganddisease.org/EN/10.14336/AD.2018.1028

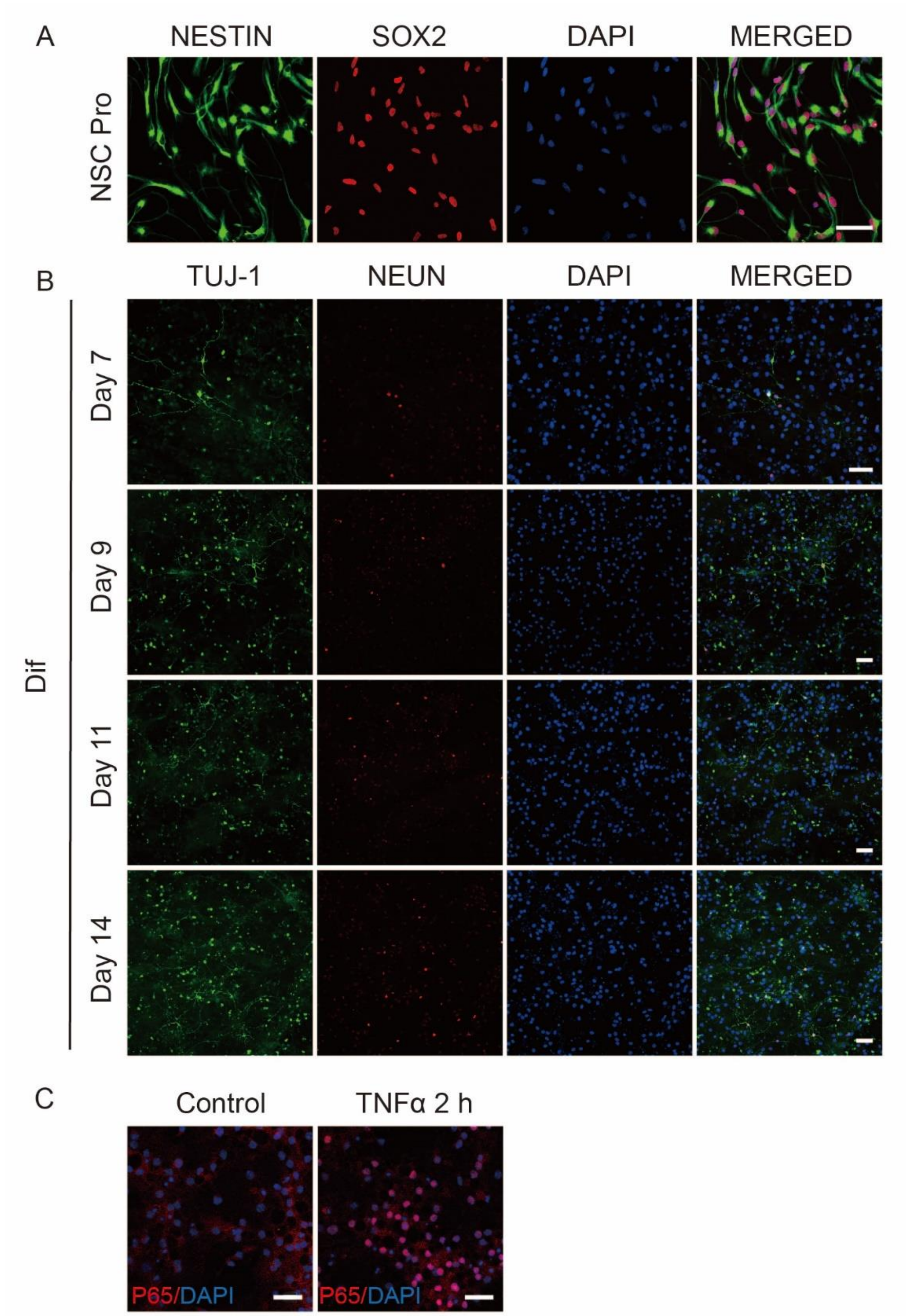

Supplementary Figure 3. Characterization of neural stem cells and activation of NF-kB pathway after TNFα treatment. (A) Monolayer murine NSCs were cultured in proliferation medium, and the cells were positive for NSC markers NESTIN and SOX2. Pro, proliferation. Scale bars, 50 μm. **(B)** Monolayer murine NSCs were subjected to a differentiation condition for 7, 9, 11 and 14 days. Early neuronal marker TUJ-1 and mature neuron marker NEUN were stained. Dif, differentiation. Scale bars, 50 μm. **(C)** Monolayer murine NSCs were treated with TNFα (20 ng/ml) for 2 h. After TNFα treatment, NF-kB translocated from cytoplasm to nucleus, as evidenced by P65 staining.

© 2018. Liu Z et al. Published online at http://www.aginganddisease.org/EN/10.14336/AD.2018.1028

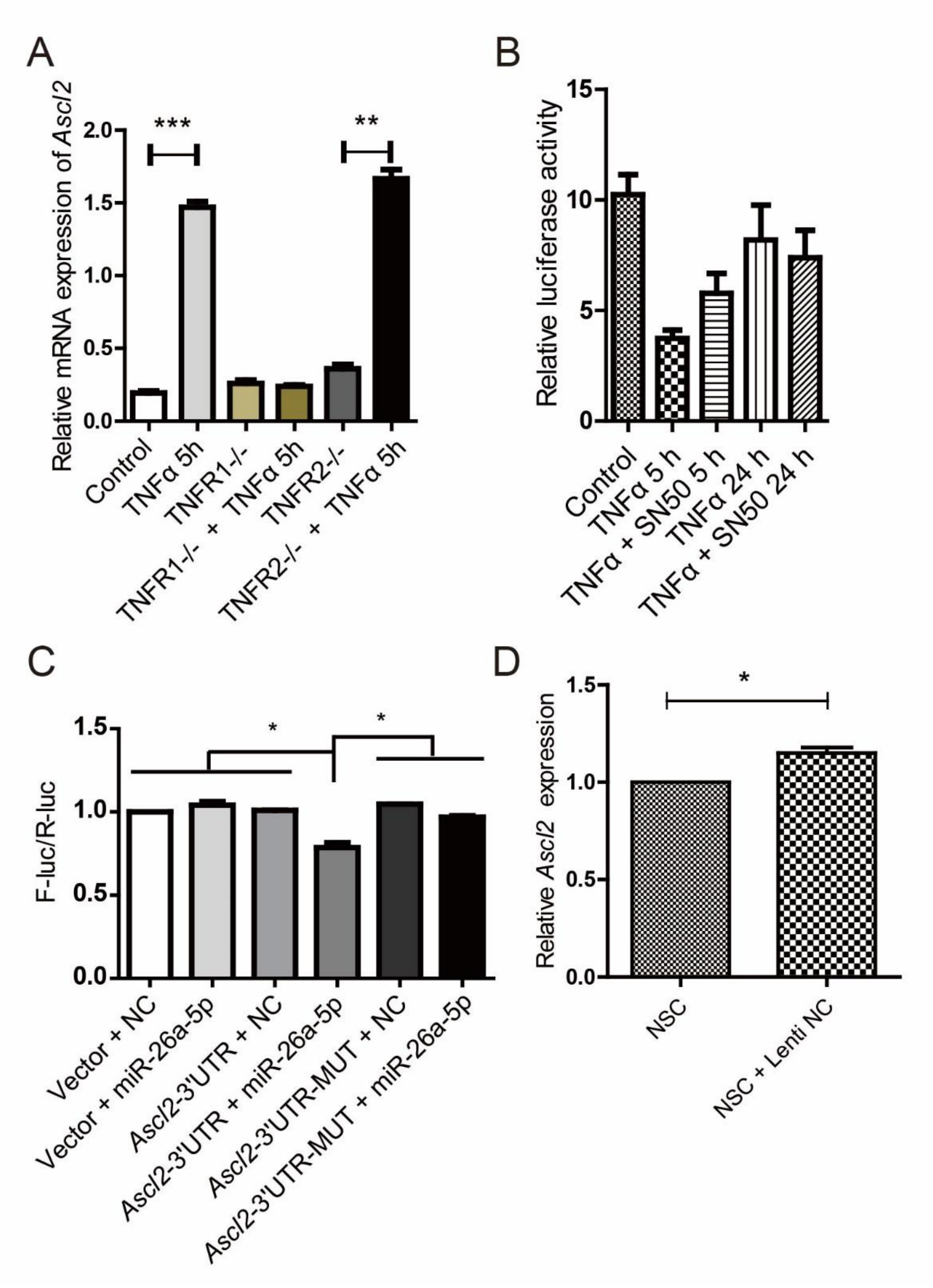

Supplementary Figure 4. Expression levels of *Ascl2* after TNFα, miRNA, and virus treatment. (A) NSCs isolated from TNFR1$^{-/-}$ and TNFR2$^{-/-}$ mice were used to determine whether TNFR1 or TNFR2 was involved in regulation of *Ascl2* by TNFα. *Ascl2* expression level analyzed by realtime PCR. (n=3). **(B)** NSCs cells were transfected with pGL3-*Ascl2* Promoter (-968 to +444bp) together with Renila vector. The next day treat cells with TNFα and/or SN50 for 5 h or 24 h. 24 h after treatment, the cells are lysed for Dual luciferase assay. Firefly luciferase activities were examined and normalized to Renilla luciferase activities. (n=3). **(C)** 293T cells were transfected with empty control vectors, *Ascl2* 3'UTR luciferase construct or *Ascl2* 3'UTR mutation luciferase construct together with miR-26a-5p mimics. Forty-eight hours following transfection, cells were collected and lysed. Firefly luciferase activities were examined and normalized to Renilla luciferase activities. (n=3). **(D)** *Ascl2* expression increased in NSCs infected with lentivirus. (n=3).

© 2018. Liu Z et al. Published online at http://www.aginganddisease.org/EN/10.14336/AD.2018.1028

SUPPLEMENTARY DATA

Ascl2 promotor sequence cloned into pGL3 vector

>NC_000073.6:142968537-142969943 Mus musculus strain C57BL/6J chromosome 7, GRCm38.p4 C57BL/6J

AGCACACTTCTGGGACAATGGGGCAAAACTGAGACTTTGACAAGGTCAGTGCATTTCC
CTTCCCCTTCAACCTTCCCTCCACCGGCCTCAGGGGAAGGATGGGACTGAGAGGTCT
GCTGGAATCCGAAGAAGTGTTATTGAACAAAAAGTCCAGGAACCGAACAAAAACTAGT
CTGGGTAGATTCACCATTAGGAAAAGAGACCCAGACTTGGAGAGCTGGCTGTAAGGTG
CAGATGGGTGACAGTGTGAGAAAGGAGTGGACTTGATTGTATTCTCTCAGGTCAGGGC
AACCGGTCCTGGGGATTCTTGAAAACACAGAGGCATGGGGTGTGCATGAGGGGCTAA
ATGGGTACAGGTGCCCAGGAA**CATGAAATTTCCCAT**TACTGGTCTGAACCACTCCTCA
AGCCCTTCTCACACTCAAGGGGCACAGGTGTGCTCCTGCAATGCGTGGACACCGGG
TCCTGGTGGAGGAGCAGGGTGGGGCGGAGTGGGCGTGGAGCCTTGTGCGAGACAC
TCCCCCATCTCTGGAGCCACGCGCCAGGCGTACGCTTCCTTCTGGCCCCGGCATAGG
ACCGCGCCAATTGTCATTGGCCAAACGGGCGATCCCAGATTGGCTGAGACCCCGGCT
CCCGCCTCCTCCGCCAGGGGGAGGGACGTGGGTGCGGGTTTGAGGCTC**G**TGCTACC
CTAGGGGGTCGCGCTCTTCTGCCTCCTACCTCTTGGTCACCGCAAAGCTTGGTCCGG
TTCTTCATCCGGCTGCAAGCGCTAGGTGTGCGGAGACCTGGCAGCTCTTGGGGCTTA
AGGGCTGAGCACCAGGACGGGTGGAGGTGCCTGTAGAGTACATTCGGACCCTCTCTC
GGACCCTCTCTCAGCCCCTGAGTGTGCGGGACCTGCGGAGCGCAGTTCGGGATCTG
CACTCGAGGATTTTTCGAGGACGCAATAAGCTAAGCATCTGCCCGGAGCATGGAAGCA
CGTCAGTAGGCCATGAACTGCACCCGGGAGGGGTGGGGGTGGAAGCGCACGGTGTC
AGCTTTGCAGAATGTGTACGCCAAGGGGAGGGTGAAGCGTGGCGGGAGGGCGAGGC
GAAGGAAGGAGGGCGTGAGAAAGGAGGCGGTGGCGGGCGGAGGAGAGTTATCTATA
CTTTTTAAAAAAAAGGAGCCGCTTGAGCCGCGTAAAGGGAGACTTGGGGAGCGCCTG
ACAGCACGCGCGGGACACGAGAGTACCACGCTTCCCTACTCTTTCAGACCTTGACTG
GTACGGGGTCCCAGGACTGCAGGAGGCCAGCGACGCGTGCCCTAGGGAGTCCTGCA
GCAGTGCCCTGCCTGAGGCCCGTGAAGGTGCAAACGTCCACTTCCCACCGCACCCG
GTTCCTCGCGAGCACTTTTCCTGTGCCGCACCAGAACTC

Predicted NFκB Binding site: **CATGAAATTTCCCAT**; Transcript start site: **G**

Supplementary Figure 5. Luciferase Reporter Assay. Software LASAGNA-Search 2.0 predicts a NF-κB binding site in *Ascl2* promotor.

© 2018. Liu Z et al. Published online at http://www.aginganddisease.org/EN/10.14336/AD.2018.1028

SUPPLEMENTARY DATA

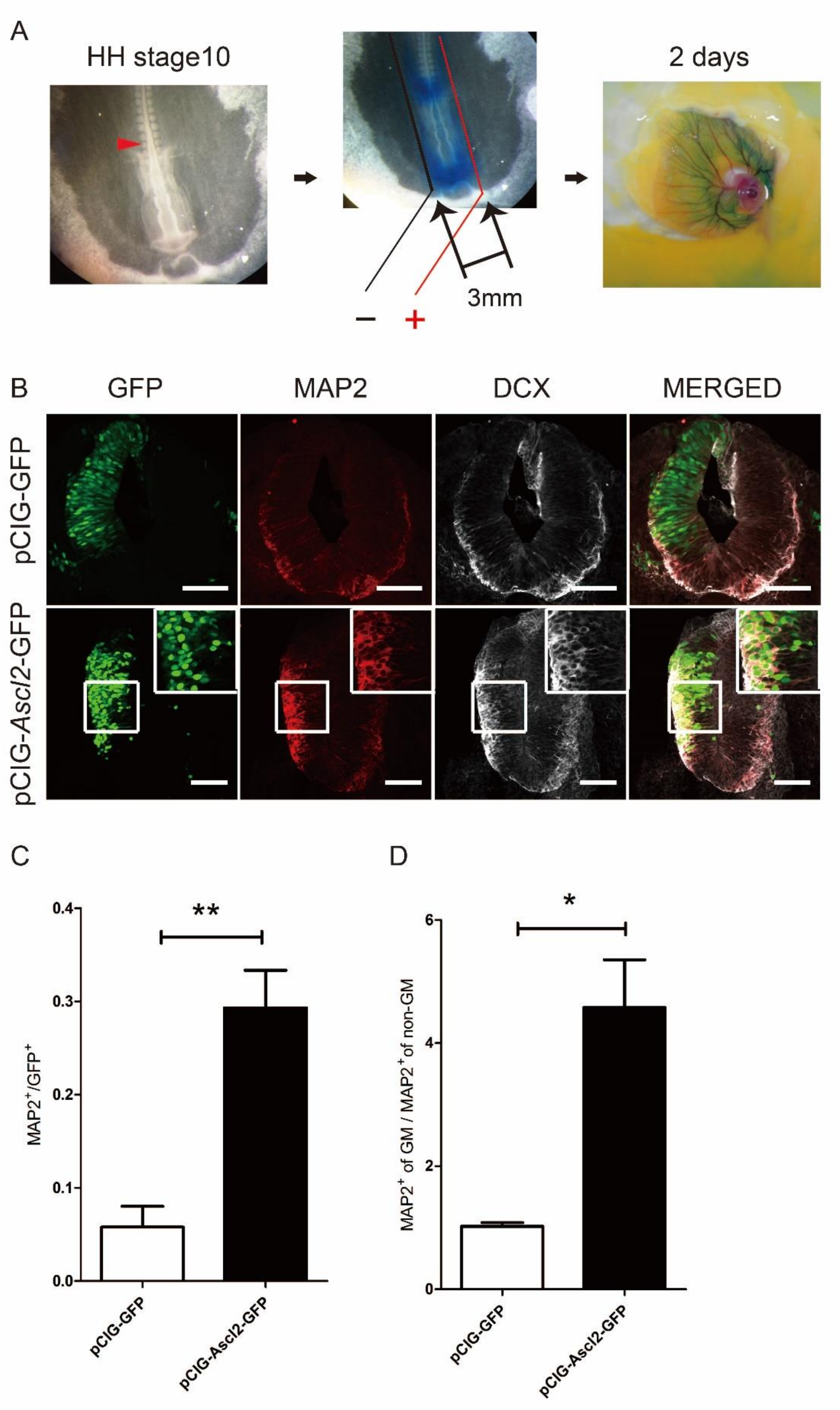

Supplementary Figure 6. The impact of *Ascl2* overexpression on NSC differentiation *in ovo*. Related to Figure 7. (A) Schematic representation of *in ovo* electroporation of *Ascl2*. **(B)** Expression of neuronal marker MAP2 at 24 h post-electroporation. **(C)** Proportion of MAP-positive cells among GFP-positive cells. (n=6). **(D)** Rate of MAP-positive cells of genetically modified (GM) side over non-genetically modified (non-GM) side. (n=6)

© 2018. Liu Z et al. Published online at http://www.aginganddisease.org/EN/10.14336/AD.2018.1028

SUPPLEMENTARY DATA

Supplementary Table 1. Primer sequences used for quantitative real time PCR. Related to Figure 1 and supplementary Figure 2.

Gene ID	Primer sequence (5' to 3')	Product Size(bp)
Gapdh: NM_138654.3	TCAACAGCAACTCCCACTCTTCCA	117
	ACCACCCTGTTGCTGTACCGTATT	
Pten: NM_008960.2	AATTCCCAGTCAGAGGCGCTATGT	137
	GATTGCAAGTTCCGCCACTGAACA	
Ascl2: NM_008554	ACCTGCTTCTAGCCCAAGAAACCT	139
	TGCAAGGTCCGGAAGATGGAAGAT	
NeuroD: NM_010894.2	ACCTTGCTACTCCAAGACCCAGAA	139
	TTTGCAGAGCGTCTGTACGAAGGA	
GFAP: NM_001131020	GGAAATTGCTGGAGGGCGAAGAAA	163
	TGGTGAGCCTGTATTGGGACAACT	
P21: NM_007669	TTGTACAAGGAGCCAGGCCAAGAT	119
	ACCCACTAAGTGCTTTGACACCCA	
Mash-1: NM_008553	ACGACTTGAACTCTATGGCGGGTT	100
	AAGTCCAGCAGCTCTTGTTCCTCT	
JHDM3A: NM_172382	GGCTTCAACCATGGCTTCAACTGT	194
	TCAATAACCGTGCTGTCCTTCCCA	
Hes6: NM_019479	TCACTGAAGCTGCTCCTCGTTTGT	80
	ATTCGGTTGGAGCATCGATGGGAT	
Ascl2 promotor: NC_000073.6	CGCGGTACCTTGAGAGCACACTTCTG	1407
	CGCGGGATCCTACGAGTTCTGGTGC	

Note: the red part indicates kpn1 and BamHI site respectively.

Supplementary Table 2. Primers and probes for miR-26a-5p Taqman quantitative real-time PCR. Related to Figure 2.

U6	FO	ATTGGAACGATACAGAGAAGATT
	RE	GGAACGCTTCACGAATTTG
	PR	TGCGCAAGGATGACACGCA
mmu-miR-26a-5p	FO	TCGCCGTTCAAGTAATCCAG
	RE	CAGAGCAGGGTCCGAGGTA
	PR	ACCCGAGAGCCTATCCTGGA
mmu-miR-26b-5p	FO	CGCCGTTCAAGTAATTCAGG
	RE	CAGAGCAGGGTCCGAGGTA
	PR	CGTTCGCTCTGGACCCGAGAC
mmu-miR-26b-3p	FO	CAGTGCTGCCTGTTCTCCATTAC
	RE	TATGGTTGTTCACGACTCCTTCAC
	PR	CCCTATCCAACCATACAGACGAGCC
mmu-miR-26a-1-3p	FO	CAGTGCTGCCTATTCTTGGTTAC
	RE	TATGGTTGTTCACGACTCCTTCAC
	PR	CCCTATCCAACCATACAGACCGTGC
mmu-miR-26a-2-3p	FO	CAGTGCTGCCTGTTCTTGATTAC
	RE	TATGGTTGTTCACGACTCCTTCAC
	PR	CCCTATCCAACCATACAGACGAAACA

Note: FO, forward primer; RE, reverse primer; PR, probe.

© 2018. Liu Z et al. Published online at http://www.aginganddisease.org/EN/10.14336/AD.2018.1028
